# Do baseline participant characteristics impact the effectiveness of a mobile health intervention for depressive symptoms? A post-hoc subgroup analysis of the CONEMO trials

**DOI:** 10.47626/1516-4446-2023-3172

**Published:** 2024-03-25

**Authors:** Heloísa Garcia Claro, Paulo Rossi Menezes, Ivan Filipe Fernandes, Nadine Seward, Juan Jaime Miranda, Maria Giovana Borges Saidel, Aline Geovanna de Lima Baquete, Kate L. Daley, Suzana Aschar, Daniela Vera Cruz, Hellen Carolina Martins Castro, Thais Rocha, Julieta Quayle, Tim J. Peters, Ricardo Araya

**Affiliations:** 1 Faculdade de Enfermagem, Universidade Estadual de Campinas, Campinas, SP, Brazil; 2 Núcleo de Apoio à Pesquisa em Saúde Mental Populacional, Universidade de São Paulo (USP), São Paulo, SP, Brazil; 3 Departamento de Medicina Preventiva, Faculdade de Medicina, USP, São Paulo, SP, Brazil; 4 Centro de Engenharia, Modelagem e Ciências Sociais Aplicadas, Universidade Federal do ABC, Santo André, SP, Brazil; 5 Centre for Global Mental Health and Primary Care Research, Health Service and Population Research, Institute of Psychiatry, Psychology, and Neuroscience, King’s College London, London, UK; 6 Bristol Medical and Dental Schools, University of Bristol, Bristol, UK

**Keywords:** Digital technology, behavioral research, depression, randomized controlled trial, mobile applications

## Abstract

**Objective:**

To ascertain whether sociodemographic and health-related characteristics known from previous research to have a substantive impact on recovery from depression modified the effect of a digital intervention designed to improve depressive symptoms (CONEMO).

**Methods:**

The CONEMO study consisted of two randomized controlled trials, one conducted in Lima, Peru, and one in São Paulo, Brazil. As a secondary trial plan analysis, mixed logistic regression was used to explore interactions between the treatment arm and subgroups of interest defined by characteristics measured before randomization – suicidal ideation, race/color, age, gender, income, type of mobile phone, alcohol misuse, tobacco use, and diabetes/hypertension – in both trials. We estimated interaction effects between the treatment group and these subgroup factors for the secondary outcomes using linear mixed regression models.

**Results:**

Increased effects of the CONEMO intervention on the primary outcome (reduction of at least 50% in depressive symptom scores at 3-month follow-up) were observed among older and wealthier participants in the Lima trial (p = 0.030 and p = 0.001, respectively).

**Conclusion:**

There was no evidence of such differential effects in São Paulo, and no evidence of impact of any other secondary outcomes in either trial.

**Clinical trial registration:**

NCT02846662 (São Paulo, Brazil – SP), NCT03026426 (Lima, Peru – LI). Funded by the U.S. National Institute of Mental Health (grant U19MH098780).

## Introduction

In low- and middle-income countries (LMICs), only 4.7% of people needing mental health care receive even “minimally adequate” services. This treatment gap needs to be addressed as a priority.^1^,^2^ Integration between psychological and physical healthcare services is paramount to ensure care and investment needs are met.^3^-^6^

Technological interventions may be a tool to address the treatment gap in these settings, and many studies indicate they are effective.^6^-^15^ There is much less evidence regarding subgroups for which such interventions may be differentially effective. Formal subgroup analyses can be used to ascertain whether subgroups modify the effects of such interventions based on demographic variables. To be adequately powered, these analyses require large sample sizes^15^,^16^ and, hence, whether pre-specified or otherwise, are essentially exploratory. This applies to the trials analyzed herein, even though they are, to our knowledge, the most extensive trials targeting depression comorbid with chronic diseases in LMIC.

CONEMO (Emotional Control in English, *Controle Emocional* in Portuguese, or *Control Emocional* in Spanish) is a low-intensity, mobile application-based intervention designed to reduce depressive symptoms. The CONEMO study comprises two trials, one conducted in São Paulo (SP), Brazil, and one conducted in Lima (LI), Peru. As part of a secondary trial plan analysis, we examined different subgroups according to baseline values in a sample of people who self-reported being treated for diabetes and hypertension and who also have depressive symptoms – Patient Health Questionnaire (PHQ-9) scores ≥ 10 – to investigate whether there is heterogeneity in the study outcomes. These analyses add to the two pre-specified subgroup analyses published in the main trial paper, covering the effects of educational attainment and baseline PHQ-9 on the main hypothesis test.^17^ The secondary data analysis reported herein assesses the impact of baseline variables on reduction of PHQ-9 scores (mean difference at least 50%) after 3 months of follow-up compared to the first wave of data collection.

Participant flow is described in the main paper.^17^ Briefly, we approached 7,597 candidates in LI. Of these, 5,785 (76.1%) accepted to be pre-screened, 787 (10.4%) fulfilled screening criteria, and 432 (5.7%) agreed to enter the study and were individually randomized either to CONEMO or enhanced usual care (EUC). In SP, we approached 11,604 candidates. Of these, 10,688 (92.1%) accepted to be pre-screened, 1,180 (10.1%) fulfilled screening criteria, and 880 (7.5%) agreed to enter the study.

## Methods

Two trials – a multicenter randomized controlled trial in LI with individual randomization and a cluster randomized controlled trial in SP with family health units as the unit of randomization – make up the CONEMO study. In SP, randomization was stratified by services with residency programs, while in LI, it was stratified by each health service and a dichotomous baseline PHQ-9 severity variable (PHQ-9 < 15 or PHQ-9 ≥ 15). In both trials, the CONEMO intervention targeted depressive symptoms in individuals with hypertension, diabetes, or both.^17^

### Ethics statement

For the SP trial, the research protocol received institutional approval on May 4, 2016 (Faculdade de Medicina, Universidade de São Paulo); national approval on May 3, 2016 (Comitê Nacional de Pesquisas, CONEP; review number 2.607.142); and municipal approval on June 3, 2016 (Secretaria Municipal de Saúde de São Paulo, the manager of all health facilities where data were collected).

In LI, the protocol was approved by six local institutional review boards: Universidad Peruana Cayetano Heredia, approval date September 8, 2016; Hospital Nacional Arzobispo Loayza, approval date September 30, 2016; Hospital Nacional Cayetano Heredia, approval date October 3, 2016; Hospital Nacional Dos de Mayo, approval date December 15, 2016; Hospital Suárez Angamos Essalud, approval date January 5, 2017; and Red Desconcentrada Sabogal Essalud, approval date April 11, 2017.

### Hypothesis

We hypothesized that there would be heterogeneity in the effectiveness of CONEMO in reducing PHQ-9 depressive symptom scores by at least 50% after 3 months of follow-up compared to the first wave of data collection, in the LI and SP arms, across subgroups of participants. The variables we tested for subgroup effects were suicidal symptoms at baseline, race/color, age, gender, income, type of mobile phone, alcohol misuse, tobacco use, and whether the participant had diabetes, hypertension, or other chronic diseases.

### Participants

We recruited participants between September 2016 and September 2017. In SP, we approached 11,604 to reach the target of 880 participants who scored at least 10 on the PHQ-9 scale, our main inclusion criterion. We only included people aged 21 or older, under treatment for diabetes and hypertension (except if gestational), who were able to read a brief text on the research assistant’s tablet.^17^

### Interventions

#### EUC group

Participants from the control group (EUC) received physical and mental health care management in family health units (in SP) and management of diabetes or hypertension (or both) in LI health services. For ethical reasons, every participant from both groups that had clinically significant depressive symptoms (PHQ-9 scores of 10 or higher) was referred back to the facility at which they were already receiving care for diabetes, hypertension, or both. Also, EUC participants were assessed for depressive symptoms throughout the study (at least four times) and referred to mental health care services when considered high-risk (PHQ-9 score ≥ 20) or at risk of suicide, according to the safety protocol.

#### CONEMO plus EUC group

CONEMO is delivered by a smartphone application supported by a nurse or nurse assistant.^18^ The application consists of 18 automated sessions, delivered over 6 weeks at a rate of three sessions per week. Information on the app’s use was captured and sent to a server where data monitoring participants’ access and progress were collected. The CONEMO group participants were also referred to treatment within their original health systems, as in the EUC group.

### Assessments

The PHQ-9 was administered in person by trained research assistants (RAs) at screening and follow-ups. We also collected data on other self-reported scales and sociodemographic parameters at baseline and after 3 months. The Suicide Risk Assessment Protocol (S-RAP) was used to measure the suicide risk of potentially eligible participants and monitor each participant’s risk longitudinally.^19^

At baseline and follow-ups, RAs assessed the participants using the PHQ-9 for depressive symptoms,^20^ the European Quality of Life, 5 Dimensions, Three Levels (EQ-5D-3L) for quality of life,^21^ the Behavioral Activation for Depression Scale-Short Form (BADS-S.F.) for behavior activation changes,^22^ and the World Health Organization Disability Assessment Schedule-II (WHODAS-II) for levels of disability.^23^ Alcohol intake was assessed using the Alcohol Use Disorders Identification Test (AUDIT-C).^19^-^24^

### Outcomes

The primary outcome is the dichotomous variable of whether the participant’s treatment was successful (a reduction in PHQ-9 score of at least 50% from baseline). We also investigated the impact of CONEMO on secondary outcomes (EQ-5D-3L, BADS-SF, WHODAS-II).

All data were collected by RAs, who also collected self-reported data on sociodemographics, diagnosis and treatment of chronic conditions (hypertension, diabetes, or both), health-services utilization (number of outpatient consultations and visits, hospitalizations reported at baseline and follow-ups, and so on), as well as some validated scales to measure specific outcomes.

### Statistical analysis

We performed all statistical analyses in Stata 15.^25^ All models have a mixed effects structure with the health unit services as random intercepts; all other variables are treated as fixed. The significance level was set to alpha = 0.05 for all statistical tests. The interaction between the treatment arm and the relevant subgroup variable and likelihood-ratio tests for the overall interaction effects are reported.

We used mixed logistic regression analyses with the dichotomous variable of success (achieved at least 50% PHQ-9 reduction at follow-up of 3 months vs. did not) as our primary outcome, and subgroup variables as explanatory variables interacting with treatment arms. Adjustments to the stratification variables used in the random sampling of treatment arms were implemented in the statistical model.

The estimated mixed logistic regression model can be written as *y_ij_* = *α_j_* + *β*_1_ *X_i_* + *δ*_1_ *S_i_* + *β*_2_ *X_i_* * *δ*_2_ *S_i_* + *λV_i_* + *e_ij_*, where *y_ij_* is the probability of treatment success, *X_i_* corresponds to the randomized treatment, and *S_i_* is the subgroup.

We performed linear mixed regression models for the secondary continuous BADS-SF, EQ-5D-3L, and WHODAS-II variables with health unit services as random intercepts, using the baseline score as a covariate, and all other variables considered as fixed.

The estimated linear mixed regression model can be written as *y_ij_* = *α_j_* + *β*_1_ *X_i_* + *δ*_1_ *S_i_* + *β*_2_ *X_i_* * *δ*_2_ *S_i_* + *φY_i_* + *λV_i_* + *e_ij_*, where *y_ij_* is the expected score of the secondary variable, *X_i_* corresponds to the randomized treatment, *S_i_* to the subgroup, and *Y_i_* is the baseline score as covariate.

For both models, V is the vector of covariates – the stratification variables – and *e_ij_* is the error term: the subscript *i* indicates the individual; *j*, the health facilities; and *α_j_*, the random-intercept by health facility.

## Results

Tables 1 to [Table t02] [Table t03] 4 present the overall (likelihood ratio) test of the interaction effects for the various outcomes and subgroup characteristics, as well as descriptive statistics plus regression coefficients of treatment effects and their 95%CIs for each subgroup.

### Subgroups

We investigated differential effects across the following subgroups: suicidal symptoms (never had/had in the past 2 weeks/had before past 2 weeks), ethnicity (white/non-white, SP only), age (up to 60 years old/60 or older), gender (female/male), household income (up to two times the minimum wage/more than two times the minimum wage, in local currency: soles in LI and real in SP), type of phone owned by the participant (not the research borrowed phone but their own: smartphone, non-smart mobile phone, or neither), alcohol misuse (AUDIT-C ≥ 2), tobacco use (yes/no), and chronic condition (diabetes, hypertension, or both). We could not analyze the effect of race in LI, since the population in Peru is distinct in many cultural aspects from that of other Latin American countries.^26^

### Participant characteristics

The control and treatment arms were balanced on several characteristics, such as gender, age, educational level, income, marital status, chronic diseases, and depression severity at baseline,^17^ as expected in randomized controlled trials such as the present studies (Table 1). Most participants were female, had a partner, and earned low incomes. Participants in LI were older and had higher educational attainment than the SP sample. Among LI participants, 185 were treated for diabetes, while 471 in SP were treated for hypertension. The severity of participants’ depressive symptoms was categorized as moderate (337 [42.39%] in SP and 275 [63.66%] in LI), moderately severe (287 [36.10%] in SP and 113 [26.16%] in LI), or severe (171 [21.51%] in SP and 44 [10.19%] in LI), with a higher proportion of severe cases in SP.^17^ In the SP trial, 334 of the 440 intervention participants (75.9%) borrowed mobile phones for the duration of the study, as did 209 of the 216 intervention participants (96.3%) in LI. The remaining participants from the intervention group either never received the research phone because they did not participate (73 [16.6%] in SP and 4 [1.8%] in LI) or returned it before the end of the intervention (33 [7.5%] in SP and 4 [1.8%] in LI).

### Subgroup analyses

The main CONEMO trials analysis^17^ showed that the intervention affected the primary outcome, i.e., the dichotomous success variable of whether the participant reached a reduction of at least 50% in PHQ-9 score at 3-month follow-up when compared to baseline values. While depressive symptoms decreased in both trial arms (CONEMO and EUC), there were between-group differences in the primary outcome. Specifically, in the CONEMO group compared with EUC, the odds ratio (OR) for successful treatment was 1.6 in SP and 2.1 in LI. There was no evidence of differential effects on the primary outcome according to educational level or baseline severity of depressive symptoms.^17^

The subgroup analyses for treatment success, reported in the present paper, found no evidence of any subgroup effects in SP (Table 1).

In LI, the effects of the intervention were stronger for older participants (interaction p = 0.030) and those in the higher income category (interaction p = 0.001). For age, the OR of treatment success was larger in participants over 60 (OR = 3.4, 95%CI 1.9-6.1) than in those under 60 (OR = 1.4, 95%CI 0.8-2.5); for income, an even greater differential effect was observed, with a much larger OR in the higher income group (OR = 8.1, 95%CI 3.4-19.3) compared with the lower income group (OR = 1.4, 95%CI 0.9-2.3) (Table 1).

Analyses of three secondary outcomes (Tables 2, 3, and 4) yielded no evidence of interaction effects for these measures, apart from an isolated finding for technology in LI (interaction p = 0.015); given the multiple tests conducted for the secondary outcomes, this observation could well be a chance finding.

## Discussion

This study examined the potential impact of nine baseline participant characteristics on the effectiveness of CONEMO, a technological intervention for depressive symptoms trialed in São Paulo, Brazil, and Lima, Peru. For the primary outcome (depressive symptoms), we found that CONEMO had subgroup effects for age and income in one of the trials (LI), with older age and higher income associated with greater intervention success. There is virtually no evidence of any interaction effects for the three secondary outcomes of interest in either of the trial sites.

To our knowledge, these are the first trials to examine the association of such recruitment variables with the effectiveness of a technological intervention for depression, quality of life, disability, behavioral activation, and service utilization.

It is essential to highlight that the interaction tests are underpowered, which means that the results of all such exploratory analyses require further investigation before being seen as robust, especially since neither of the two prespecified subgroup analyses yielded evidence of differential effects.

We found the effects of CONEMO to be stronger in LI for older participants (OR = 3.4, 95%CI 1.9-6, interaction p-value = 0.030) than in those under 60 (OR = 1.4, 95%CI 0.8-2.5), and among participants in the higher income category (OR = 8.1, 95%CI 3.4-19.3) compared to those in the lower income group (OR = 1.4, 95%CI 0.9-2.3, interaction p = 0.001). Previous studies using digital gamification interventions focused on reducing depressive symptoms and improving well-being also encountered better effectiveness among older adults when compared to younger people or the general population in subgroup analyses of a systematic review and meta-analysis.^27^ This result contradicts the common-sense hypothesis that older people do not benefit from digitally facilitated treatment. The literature also provides evidence that older adults are often satisfied and report well-being and perceived changes in several outcomes related to mental health after interacting with digital interventions.^28^

Regarding income interaction, several baseline features – including income – have been reported to be associated with higher depression remission rates in other studies. Such studies conclude that socioeconomic status can impact health outcomes, but further investigation of these interactions is needed.^29^-^31^ Randomized controlled trials often find an interaction (or at least a suggestion of one) between depression severity and the success of interventions.^26^,^32^-^34^ Indeed, we found a lower effect in participants with severe symptoms in LI and for participants with a lifetime history of suicidal ideation in LI, as also observed in other studies.^35^,^36^ These results suggest that those in LI with severe symptoms, both in terms of depression scores and history of suicidal ideation, respond less to treatment than participants with less severe symptoms.

As observed in other studies, white people in SP,^37^ people without smartphones in SP or no mobile phone at all in LI,^38^ smokers,^39^ people at risk of harmful alcohol consumption in SP,^40^ people diagnosed with both hypertension and diabetes in SP, and those diagnosed with only diabetes in LI^40^,^41^ appeared to have no success related to the intervention. However, we could not detect effects of the interactions corresponding to these suggestions in our sample sizes for the trials presented herein.

Further studies are needed to confirm these null results.

When subgroup analyses are not well justified from the literature and established expectations, they may yield misleading conclusions. This study proposed interaction analyses with variables well established in the literature as potentially influencing the effects of interventions designed to relieve depressive symptoms. However, these results are limited to the CONEMO intervention as trialed in SP and LI.

Our initial hypothesis of heterogeneous effects regarding the effectiveness of CONEMO in improving depressive symptoms in participants among the various subgroups was largely unconfirmed.

Taken together, these findings allow us to conclude that the CONEMO intervention can be effective for most subgroups studied. However, additional replications with participants who use tobacco or other drugs or belong to different subgroups would be desirable, as would studies designed to investigate differential and interaction effects.

In trials of technological interventions such as this, promoting digital access and technological literacy is vital to optimize potential benefits. Technological interventions can increase access to treatment, and studies that help provide a deeper understanding of their effects should be encouraged.

Development and improvement of technological interventions need evidence-based information. To obtain such information, more clinical trials, systematic reviews, and meta-analyses of the effectiveness of these interventions in the general population – including interaction analyses – are needed; their results can help tailor technological interventions for different groups.

Although our overall sample size was large compared to that of similar studies, some subgroups (such as tobacco users) were small. Thus, our results should be considered carefully, and other studies examining the interaction of tobacco, alcohol, and drug use with the outcome of technological interventions are needed. Indeed, the power to detect interactions from studies designed to detect overall intervention effects is likely low.^16^ Hence, the absence of evidence of differential effects in general in this study; the few interactions we observed need to be treated with caution in exploratory analyses.

Additionally, we could not analyze the effect of race in LI, since the population of Peru is distinct in many cultural aspects from those of other Latin American countries.^26^ Potential effects should be analyzed carefully, considering cultural and historical elements not addressed in this paper.^26^

## Disclosure

The authors report no conflicts of interest.

## Figures and Tables

**Table 1:** Descriptive statistics, subgroup-specific (adjusted) ORs with 95%CIs, and interaction likelihood-ratio tests for differential effects of various baseline characteristics on the primary outcome (reduction of at least 50% in PHQ-9 score at 3 months after inclusion) for each of the two trials

	São Paulo				Lima			
	Digital intervention†	EUC†	Subgroup OR (95%CI)‡	Interaction p-value§	Digital intervention†	EUC†	OR (95%CI)‡	Interaction p-value§
Suicidal ideation								
Never	300 (68)	300 (68)	1.54 (1.09 to 2.20)		120 (55)	124 (58)	2.64 (1.53 to 4.57)	
More than 2 weeks ago	85 (19)	80 (18)	2.18 (1.05 to 4.54)		79 (36)	61 (28)	1.89 (0.93 to 3.87)	
In the past 2 weeks	55 (13)	60 (14)	2.52 (1.03 to 6.15)	0.482	18 (8)	30 (14)	1.85 (0.51 to 6.69)	0.728
Ethnicity								
Non-white	305 (69)	308 (70)	1.79 (1.26 to 2.56)					
White	135 (31)	130 (30)	1.57 (0.91 to 2.72)	0.693				
Age (years)								
Younger than 60	264 (60)	269 (61)	1.62 (1.11 to 2.37)		104 (48)	100 (47)	1.39 (0.77 to 2.49)	
60 or older	176 (40)	171 (39)	1.87 (1.16 to 3.00)	0.545	113 (52)	115 (53)	3.44 (1.93 to 6.11)	0.030
Gender								
Female	378 (86)	383 (87)	1.56 (1.14 to 2.15)		186 (86)	166 (77)	2.07 (1.32 to 3.24)	
Male	62 (14)	57 (13)	2.98 (1.32 to 6.70)	0.144	31 (14)	49 (23)	3.05 (1.15 to 8.07)	0.475
Technology								
No mobile phone	82 (19)	83 (19)	3.38 (1.64 to 6.97)		29 (13)	29 (13)	2.47 (0.79 to 7.74)	
No smartphone	111 (25)	103 (23)	1.09 (0.59 to 2.01)		81 (37)	76 (35)	2.02 (1.02 to 3.97)	
Smartphone	247 (56)	254 (58)	1.67 (1.13 to 2.46)	0.060	107 (49)	110 (51)	2.36 (1.33 to 4.19)	0.926
Tobacco use								
Nonsmoker	376 (85)	361 (82)	1.81 (1.31 to 2.50)		208 (96)	208 (97)	2.26 (1.49 to 3.42)	
Smoker	64 (15)	79 (18)	1.30 (0.62 to 2.72)	0.424	9 (4)	7 (3)	2.16 (0.15 to 31.73)	0.975
Alcohol misuse								
No risk	373 (85)	380 (86)	1.80 (1.30 to 2.48)		197 (91)	188 (87)	2.10 (1.36 to 3.22)	
Risk	66 (15)	60 (14)	1.33 (0.61 to 2.88)	0.483	20 (9)	27 (13)	3.66 (1.01 to 13.29)	0.417
Missing	1 (1)	-	-		-	-	-	
Chronic conditions								
Hypertension	240 (55)	231 (53)	1.68 (1.13 to 2.51)		57 (26)	64 (30)	3.62 (1.64 to 8.01)	
Diabetes	46 (10)	44 (10)	2.71 (1.03 to 7.11)		87 (40)	98 (46)	1.53 (0.82 to 2.86)	
Both	154 (35)	165 (38)	1.54 (0.93 to 2.55)	0.588	73 (34)	53 (25)	2.53 (1.17 to 5.46)	0.231
Income								
Less than two MW	304 (69)	293 (67)	1.83 (1.27 to 2.62)		152 (70)	148 (69)	1.44 (0.89 to 2.31)	
Two or more MW	132 (30)	141 (32)	1.53 (0.90 to 2.58)	0.582	64 (29)	64 (30)	8.07 (3.38 to 19.26)	0.001
Missing	4 (1)	6 (1)	-		1 (1)	-	-	

Data presented as n (%), unless otherwise specified.

EUC = enhanced usual care; MW = minimum wage; OR = odds ratio; PHQ-9 = Patient Health Questionnaire.

† Descriptive statistics for each subgroup by trial intervention (digital intervention and EUC).

‡ Estimated OR of the primary outcome for each subgroup with respective 95%CI from the relevant random-effects logistic regression model adjusting for stratification and (for São Paulo) clustering by primary care unit as the unit of randomization.

§ p-values derived for the interaction terms (intervention effect by baseline characteristic) by the likelihood ratio F-test in the random-effects regression model.

**Table 2 t02:** Subgroup-specific (adjusted) differences in means with 95%CIs and interaction tests for differential effects of various baseline characteristics on the secondary outcome continuous BADS-SF scores at 3 months after inclusion, for each of the two trials

	São Paulo		Lima	
BADS-SF†	Difference in means (95%CI)‡	Interaction p-value§	Difference in means (95%CI)‡	Interaction p-value§
Suicidal ideation				
Never	1.15 (-0.15 to 2.44)		2.41 (0.27 to 4.55)	0.371
More than 2 weeks ago	0.32 (-2.20 to 2.84)		4.89 (2.07 to 7.72)	
In the past 2 weeks	1.47 (-1.48 to 4.41)	0.811	4.27 (-0.84 to 9.39)	
Ethnicity				
Non-white	0.94 (-0.36 to 2.23)		-	
White	0.96 (-1.03 to 2.95)	0.985	-	-
Age (years)				
Younger than 60	1.09 (-0.27 to 2.45)		4.45 (2.08 to 6.83)	
60 or older	0.73 (-1.06 to 2.52)	0.755	2.99 (0.76 to 5.22)	0.380
Gender				
Female	1.18 (0.01 to 2.35)		3.50 (1.70 to 5.29)	
Male	-0.46 (-3.43 to 2.51)	0.313	4.28 (0.40 to 8.16)	0.719
Technology				
No mobile phone	-0.12 (-2.69 to 2.44)		-0.55 (-5.08 to 3.98)	
No smartphone	2.12 (-0.14 to 4.38)		4.21 (1.55 to 6.87)	
Smartphone	0.86 (-0.55 to 2.28)	0.424	4.35 (2.07 to 6.64)	0.149
Tobacco use				
Nonsmoker	1.15 (-0.04 to 2.33)		3.72 (2.07 to 5.38)	
Smoker	-0.29 (-2.97 to 2.39)	0.336	2.12 (-6.45 to 10.69)	0.719
Alcohol misuse				
No risk	0.79 (-0.39 to 1.96)		3.74 (2.02 to 5.45)	
Risk	1.94 (-0.93 to 4.80)	0.466	2.70 (-2.38 to 7.78)	0.705
Chronic conditions				
Hypertension	1.68 (0.20 to 3.17)		4.77 (1.71 to 7.83)	
Diabetes	1.43 (-1.89 to 4.69)		2.64 (0.12 to 5.17)	
Both	-0.10 (-1.90 to 1.70)	0.311	3.66 (0.66 to 6.67)	0.578
Income				
Less than two MW	1.28 (-0.03 to 2.60)		3.38 (1.44 to 5.32)	
Two or more MW	0.45 (-1.50 to 2.40)	0.489	4.39 (1.33 to 7.46)	0.584

BADS-SF = Behavioral Activation for Depression Scale - Short Form; MW = minimum wage.

† BADS-SF scores range from 0 to 54; higher scores represent a higher level of activation.

‡ Estimated difference in means for intervention for each subgroup with respective 95%CI, from the relevant random-effects logistic regression model adjusting for stratification and (for São Paulo) clustering by primary care unit as the unit of randomization.

§ p-values derived for the interaction terms (intervention effect by baseline characteristic) by the likelihood ratio F-test in the random-effects regression model.

**Table 3 t03:** Subgroup-specific (adjusted) differences in means with 95%CIs and interaction tests for differential effects of various baseline characteristics on the secondary outcome continuous WHODAS-II scores at 3 months after inclusion, for each of the two trials

	São Paulo		Lima	
WHODAS-I†	Difference in means (95%CI)‡	Interaction p-value§	Difference in means (95%CI)‡	Interaction p-value§
Suicidal ideation				
Never	-2.14 (-4.60 to 0.31)		-6.46 (-10.40 to -2.53)	
More than 2 weeks ago	-2.00 (-6.83 to 2.82)		-5.04 (-10.29 to 0.21)	
In the past 2 weeks	-8.66 (-14.38 to -2.92)	0.112	-12.37 (-21.80 to -2.94)	0.411
Ethnicity				
Non-white	-1.58 (-4.07 to 0.90)			
White	-5.89 (-9.70 to -2.08)	0.063		
Age (years)				
Younger than 60	-2.90 (-5.57 to -0.23)		-8.02 (-12.34 to -3.70)	
60 or older	-2.75 (-6.13 to 0.63)	0.946	-5.70 (-9.81 to -1.59)	0.447
Gender				
Female	-2.45 (-4.69 to -0.21)		-6.34 (-9.64 to -3.04)	
Male	-5.39 (-11.05 to 0.27)	0.341||	-8.86 (-15.90 to -1.83)	0.525
Technology				
No mobile phone	-3.28 (-8.15 to 1.60)		-1.31 (-9.56 to 6.95)	
No smartphone	-2.98 (-7.29 to 1.33)		-10.55 (-15.42 to -5.67)	
Smartphone	-2.61 (-5.31 to 0.08)	0.970	-5.60 (-9.78 to -1.42)	0.119
Tobacco use				
Nonsmoker	-3.22 (-5.47 to -0.97)		-6.68 (-9.70 to -3.66)	
Smoker	-0.85 (-6.13 to 4.42)	0.419	-10.85 (-26.98 to 5.29)	0.619
Alcohol misuse				
No risk	-2.67 (-4.97 to -0.37)		-6.69 (-9.85 to -3.54)	
Risk	-2.87 (-8.37 to 2.64)	0.948	-7.77 (-16.96 to 1.42)	0.829
Chronic conditions				
Hypertension	-3.81 (-6.60 to -1.01)		-8.19 (-13.84 to -2.54)	
Diabetes	-4.15 (-10.81 to 2.52)		-4.18 (-8.79 to 0.44)	
Both	-1.08 (-4.55 to 2.39)	0.450	-9.22 (-14.75 to -3.70)	0.332
Income				
Less than two MW	-2.87 (-5.39 to -0.34)		-6.70 (-10.27 to -3.12)	
Two or more MW	-3.09 (-6.82 to 0.63)	0.922	-7.08 (-12.72 to -1.44)	0.911

MW = minimum wage; WHODAS-II = World Health Organization Disability Assessment Schedule-II.

† WHODAS-II scores range from 0 to 100; higher scores represent more severe disability.

‡ Estimated difference in means for intervention for each subgroup with respective 95%CI from the relevant random-effects logistic regression model, adjusting for stratification and (for São Paulo) clustering by primary care unit as the unit of randomization.

§ p-values derived for the interaction terms (intervention effect by baseline characteristic) by the likelihood ratio F-test in the random-effects regression model.

|| Estimated by a fixed-effects linear model.

**Table 4:** Subgroup-specific (adjusted) differences in means with 95%CIs and interaction tests for differential effects of various baseline characteristics on the secondary outcome continuous EQ-5D-3L scores at 3 months after inclusion, for each of the two trials

	São Paulo		Lima	
EQ-5D-3L†	Difference in means (95%CI)‡	Interaction p-value§	Difference in means (95%CI)‡	Interaction p-value§
Suicidal ideation				
Never	0.03 (0.01 to 0.06)		0.03 (-0.02 to 0.08)	
More than 2 weeks ago	0.06 (0.01 to 0.10)		0.06 (-0.01 to 0.12)	
In the past 2 weeks	0.02 (-0.03 to 0.08)	0.641	0.11 (-0.01 to 0.24)	0.465
Ethnicity				
Non-white	0.03 (0.00 to 0.05)			
White	0.04 (0.00 to 0.08)	0.620		
Age (years)				
Younger than 60	0.04 (0.01 to 0.07)		0.06 (0.01 to 0.12)	
60 or older	0.02 (-0.02 to 0.05)	0.348	0.05 (-0.01 to 0.10)	0.685
Gender				
Female	0.03 (0.01 to 0.05)		0.04 (-0.01 to 0.08)	
Male	0.03 (-0.03 to 0.09)	0.983	0.13 (0.04 to 0.22)	0.064
Technology				
No mobile phone	0.08 (0.03 to 0.13)		-0.08 (-0.19 to 0.02)	
No smartphone	0.02 (-0.03 to 0.06)		0.10 (0.04 to 0.16)	
Smartphone	0.02 (-0.00 to 0.05)	0.138	0.05 (-0.00 to 0.11)	0.015
Tobacco use				
Nonsmoker	0.03 (0.01 to 0.05)		0.05 (0.02 to 0.09)	
Smoker	0.04 (-0.02 to 0.09)	0.787	0.00 (-0.20 to 0.20)	0.604
Alcohol misuse				
No risk	0.03 (0.01 to 0.06)		0.05 (0.01 to 0.09)	
Risk	0.01 (-0.05 to 0.07)	0.430	0.06 (-0.06 to 0.18)	0.901
Chronic conditions				
Hypertension	0.04 (0.02 to 0.07)		0.05 (-0.02 to 0.13)	
Diabetes	0.05 (-0.02 to 0.12)		0.04 (-0.02 to 0.09)	
Both	0.02 (-0.02 to 0.05)	0.498	0.07 (0.00 to 0.14)	0.773
Income				
Less than 2 MW	0.04 (0.01 to 0.07)		0.05 (0.00 to 0.09)	
Two or more MW	0.02 (-0.02 to 0.06)	0.322	0.06 (-0.01 to 0.13)	0.772

EQ-5D-3L = European Quality of Life, 5 Dimensions, Three Levels; MW = minimum wage.

† EQ-5D-3L scores range from 0 to 1; higher scores represent better quality of life.

‡ Estimated difference in means for intervention for each subgroup with respective 95%CIs from the relevant random-effects logistic regression model, adjusting for stratification and (for São Paulo) clustering by primary care unit as the unit of randomization.

§ p-values derived for the interaction terms (intervention effect by baseline characteristic) by the likelihood ratio F-test in the random-effects regression model.

## Data Availability

We will share the data we used to calculate the results we present in this paper, without identifying information,^42^ after five years of dataset closure (January 2025) through the National Institute of Mental Health Data Archive (NDA) platform for an indefinite time. We will also provide data dictionaries, research protocols, statistical analysis plans, and data analysis codes.
